# Modulating NMDA receptors to treat MK-801-induced schizophrenic cognition deficit: effects of clozapine combining with PQQ treatment and possible mechanisms of action

**DOI:** 10.1186/s12888-020-02509-z

**Published:** 2020-03-06

**Authors:** Xingqin Zhou, Gangming Cai, Shishi Mao, Dong Xu, Xijie Xu, Rongjun Zhang, Zhiwen Yao

**Affiliations:** 1 Key Laboratory of Nuclear Medicine, Ministry of Health, Jiangsu Key Laboratory of Molecular Nuclear Medicine, Jiangsu Institute of Nuclear Medicine, Wuxi, Jiangsu Province 214063 PR China; 2 Department of Neurology, Yangpu Hospital, Tongji University School of Medicine, Shanghai, 200090 PR China

**Keywords:** Schizophrenic cognition deficit, NMDA receptor, GSK-3β/Akt signaling pathway, Apoptosis, PQQ combined with clozapine

## Abstract

**Background:**

Clozapine has remarkable efficacy on both negative and cognitive symptoms of schizophrenia due to its slight activation of NMDA receptor. In fact, much evidence to the contrary. NMDAR is a complex containing specific binding sites, which are regulated to improve negative symptoms and cognitive deficits associated with individuals affected by schizophrenia.

PQQ is a powerful neuroprotectant that specifically binds with NMDA receptors in the brain to produce beneficial physiological and cognitive outcomes. The aim of this study was to enhance NMDAR function and improve cognitive ability in schizophrenia by PQQ combined with clozapine.

**Methods:**

Rats were divided into four groups (*n* = 5) including control (saline), model (MK-801, 0.5 mg·kg^− 1^·d^− 1^), atypical antipsychotic (MK-801 (0.5 mg·kg^− 1^·d^− 1^) + Clozapine (1.0 mg·kg^− 1^·d^− 1^), and co-agonist NMDA receptor (MK-801 (0.5 mg·kg^− 1^·d^− 1^) + Clozapine (0.5 mg·kg^− 1^·d^− 1^) + PQQ (1.0 μg·kg^− 1^·d^− 1^) group. Each group of rats was injected subcutaneously every day for 6 weeks. Behavior test, including stereotyped behavior, locomotor hyperactivity, learning and memory, was performed. The Western blot assay was performed to analyze the expression of GSK-3β, Akt, NMDAR1, and MGLUR in rat hippocampus.

**Results:**

Results indicated that clozapine and PQQ combination therapy can improve MK801-induced schizophrenia behavior including stereotyped behavior, locomotor hyperactivity and cognitive impairment. Furthermore, we found that modulating NMDA receptors could ameliorate the memory impairments in Mk-801 induced schizophrenia rats by reducing the expression of NMDAR1 and MGLUR3, decreasing hippocampal tau hyperphosphorylation and inhibiting apoptosis through Akt /GSK-3β signaling pathway.

**Conclusions:**

These findings suggest that combination therapy for enhancing NMDA receptors may be able to rescue cognition deficit in schizophrenia. More studies are needed to better elucidate these mechanisms.

## Background

Schizophrenia is a complex psychiatric disorder that has been for a long time associated with dopaminergic dysfunctions in the pathological processes [1–4]. Although Dopamine Hypothesis remains dominant in the explanations of schizophrenia, it does not fully interpret the complexities and diversities of the disorder [5–7]. More exactly, glutamate and dopamine confusion are involved in the development of schizophrenia and the abnormal expression of NMDA receptors is thought to contribute to negative symptoms and cognitive deficits [8]. Up to now, cognitive impairment remains an unresolved main problem in schizophrenic patients [9]. Changes in NMDA-R expression may have a different modality depending on the applied neuroleptics. In this paper, we attend to improve cognitive ability by enhancing NMDA receptor function in the rat model of schizophrenia induced by MK-801.

Clozapine, an atypical antipsychotic agent, has mild agonistic actions on NMDA receptors [10]. Furthermore, several lines of evidence suggest that the NMDAR is involved in the antipsychotic efficacy of clozapine [11–13]. Previous findings indicate that the long-term administration of clozapine decrease the expression of NMDAR in hippocampus [14]. Clozapine with its remarkable efficacy on both negative and cognitive symptoms is considered the most effective antipsychotic drug in the treatment of people diagnosed with schizophrenia [15]. However, some patients with schizophrenia does not respond to clozapine therapy [16]. In fact, much evidence to the contrary. For these reasons, it is necessary to improve the binding of NMDAR for novel interventions.

PQQ is a redox cofactor which has been found in various vegetable and animal tissues [17, 18]. PQQ is a powerful neuroprotectant that specifically binds with NMDA receptors in brain to inhibit glutamate-induced cytotoxicity and produce beneficial physiological and cognitive outcomes [19]. NMDA receptor is a complex containing specific binding sites, which are regulated to improve negative symptoms and cognitive deficits associated with individuals affected by schizophrenia [20–22]. Our previous study found that the co-agonist NMDA receptor treated with PQQ and d-serine enhances social memory in MK-801 induced schizophrenic model [23]. If these co-agonist NMDA receptor treatments indeed target different mechanisms, the combination of PQQ and Clozapine may provide a superior therapeutic benefit. To test the above hypotheses, we have induced schizophrenic model rats using MK-801 and then probed the efficacy of NMDA (or MGLURs) receptor modulators by PQQ administration add to Clozapine. This method of improving cognitive ability through co agonist NMDA receptor will provide experimental basis for expanding the potential clinical application of clozapine in the treatment of individuals affected by schizophrenia.

## Methods

### Subjects

These experiments were conducted in male Sprague-Dawley rats (weight 280–300 g, age 6–8 weeks), provided by Changzhou Cavens Laboratory Animal Co., Ltd. (experimental animal production license: SYXK Jiangsu 2019–0025) and raised in a standard animal laboratory. The animals were housed in a climate-controlled room maintained on a 12-h light/dark cycle (lights on at 7:00 A.M.), temperature (25 ± 1 °C) and relative humidity (50–70%). Food and water were available ad libitum. Rats were divided into four groups with 5 rats per group, including control (saline), model (MK-801, 0.5 mg·kg^− 1^·d^− 1^), atypical antipsychotic (MK-801 (0.5 mg·kg^− 1^·d^− 1^) + Clozapine (1.0 mg·kg^− 1^·d^− 1^), and co-agonist NMDA receptor (MK-801 (0.5 mg·kg^− 1^·d^− 1^) + Clozapine (0.5 mg·kg^− 1^·d^− 1^) + PQQ (1.0 μg·kg^− 1^·d^− 1^) group. Each group of rats was injected subcutaneously every day for 6 weeks. All experiments were approved by the Animal Care and Ethics Committee of Jiangsu Institute of Nuclear Medicine (2016–009) and carried out according to the National Institute of Health and Guide for the Care and Use of Laboratory Animals (NIH Publications No. 85–23). Efforts were made to minimize the number of animals used and their suffering. Rats were deeply anesthetized for euthanasia by isoflurane (5%) inhalation for 2 min and quickly decapitated.

### Stereotyped behavior test

Stereotypic behavior is a typical symptom of schizophrenia, which is characterized by repeated aimless behavior, including head-weaving, turning, and rotating [24, 25]. Stereotypical behavior was measured as the total amount of time spent engaged in head-weaving, circling, and axial turning. According to the degree of stereotyped behavior, they were classified into five grades (0, 1, 2, 3 and 4), representing absent, equivocal, present, intense and continuous, respectively. Scores were recorded every 10 min for 1.5 h.

### Morris water maze test

After drug administration for 42 days, the spatial memory ability was determined using Morris water maze test (MWM). The test was performed in a black circular pool with a diameter of 180 cm and filled with water (25 °C ± 2) until 8 cm from the top. A round platform was placed 1 cm below the water surface in the center of the target quadrant. Rats were underwent two trials for 5 days consecutively with an intertrial interval of 4 h. Their escape latencies were recorded. Once the rat located the platform, it was allowed to remain on it for 10 s. If the rat did not reach the platform within 60 s, it was placed and left on the platform for 10 s and the escape latency was recorded as 120 s. After 5 days of training, the platform was removed for the probe test and each rat was allowed to swim freely for 120 s. The swimming track was recorded and the number of crossing the platform was measured.

### Open-field test

Following the MWM test, individual rats were subjected to the open field test using OFT-100 Opening Activity System (TM-Vision, Chengdu). Their various behaviors (motion and stationary) were recorded and the behavioral parameters of the experimental animals in each delimited area were analyzed using the computer vision correlation algorithm. The motion distance and trajectory was evaluated 5 times and 3 min each time.

### Western blot analysis

The Western blot assay was performed as previously described [26, 27]. Briefly, the hippocampus was homogenized in lysis buffer (Beyotime Biotechnology, Haimen, China) and centrifuged at 12,000 rpm for 10 min at 4 °C. The concentration of protein was measured using BCA assay kit (Sangon, Shanghai, China). The concentration of separation gel was selected according to the molecular weight of protein. Proteins were transferred to nitrate PVDF membranes (Beyotime Biotechnology, Haimen, China) at 350 mA for 1.5 h using a cooling system. PVDF membrane was then treated with 5% non-fat dry milk powder in Tris buffer (pH 7.4) containing 0.1% Tween-20 (TBS-T) for 1 h.

The primary antibodies in TBS-T over night at 4 °C as follows:β-actin (1:4000, Bioss, China); GSK-3β; p-GSK-3β; Akt; p-Akt; BAX; BCL-2; p-Tau; NMDAR1; MGLUR1; MGLUR2; MGLUR3 (1:400, Bioss, China). Membranes were then washed three times with TBS-T for 10 min per wash. Each membrane was incubated with the secondary antibodies for 1 h (1:5000, Bioss, China) and washed three times with TBS-T for 15 min. β-actin was used as the loading reference for data analysis. Finally, the protein bands were visualized using an enhanced ECL kit (Pierce Chemical Company, USA) and a Gel Imaging System (ChemoDOC™ XRS^+^, Bio-Rad, USA).

### Statistical analysis

The tests were analyzed using SAS 9.3. Data are shown as the mean ± standard error of the mean (SEM). A group test statistic for the equality of means is reported for both equal and unequal variances. Both the pooled test and the Satterthwaite test indicate evidence for significant levels. A *P* value of < 0.05 was considered statistically significant.

## Results

### Stereotyped behavior test

**Fig. 1:**
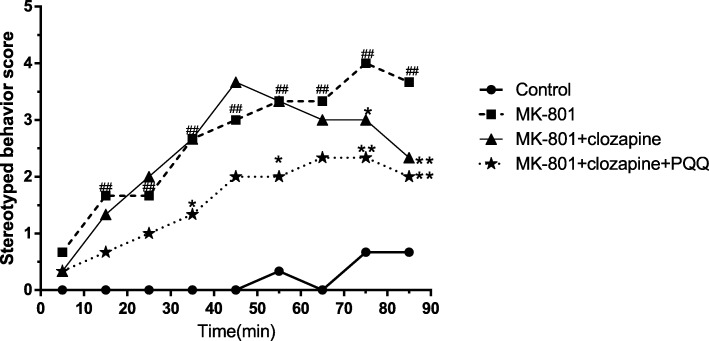
Stereotypical behavior test. ^#^*P* < 0.05 and ^##^*P* < 0.01 relative to control group. *P < 0.05 and **P < 0.01 relative to the MK-801-induced model

From Fig. 1, it can be seen that rats in the MK-801 group showed significant stereotypical behavior during the beginning of the test. The rats in clozapine group had no significant difference between 0 and 70 min compared with the MK-801-induced rats. 70 min later, the rats treated with clozapine showed fewer stereotypes. However, rats treated with clozapine and PQQ exhibited significantly less stereotypical behavior compared with rats in the MK-801 group across the entire test. The results suggested that PQQ combine with clozapine can effectively inhibit stereotyping induced by MK-801.

### Morris water maze test

**Fig. 2:**
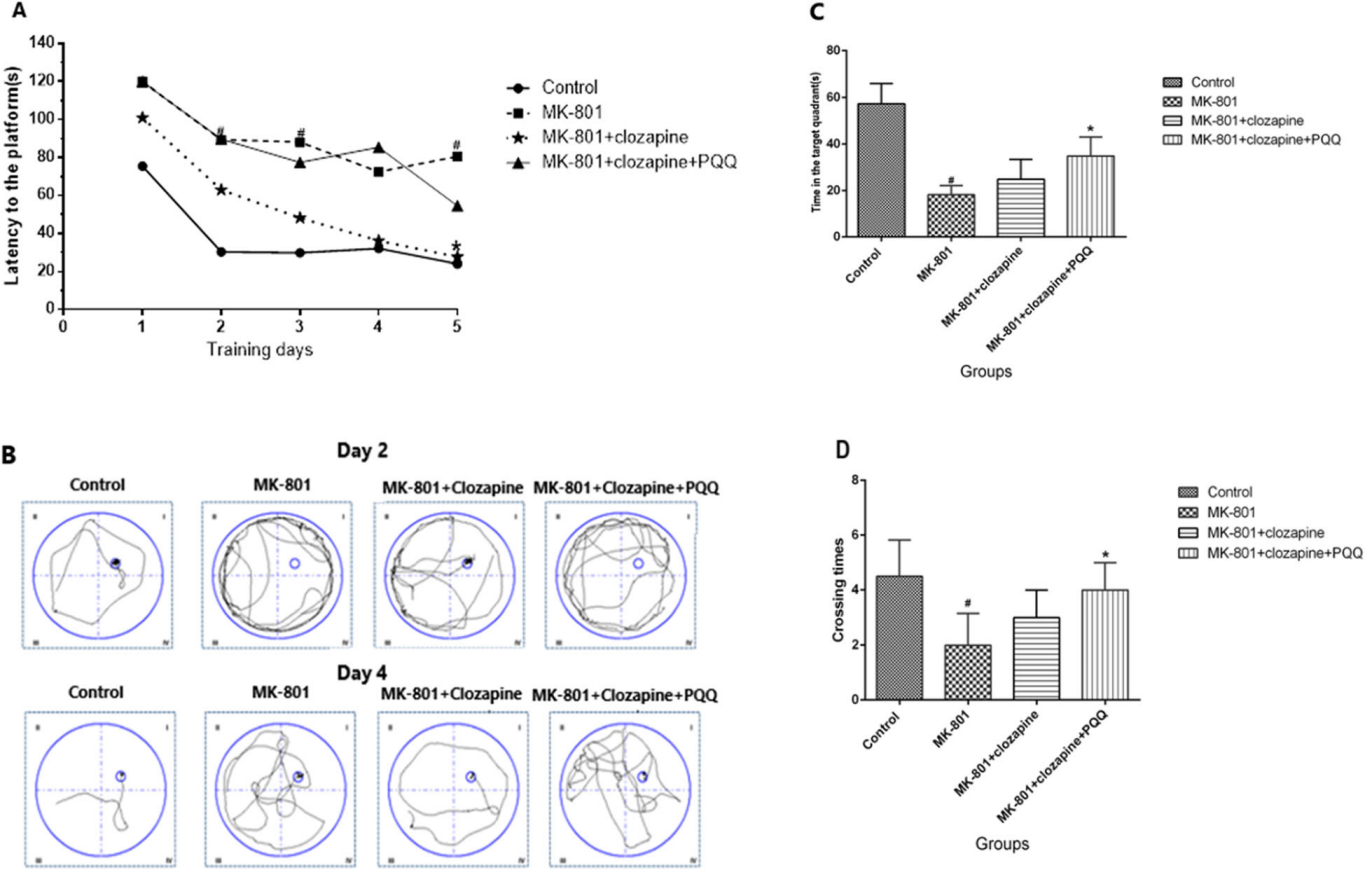
The spatial learning and memory ability test of rats using Morris water maze **a** The latencies to find a hidden platform in the water maze during five training consecutive days. **b** The swim path of rats on the 2nd d and 5th d in search strategy trails. **c** The time in the target quadrant and **d** the number of crossing times on the target platform within 120 s. ^#^*p* < 0.05 ^##^*p* < 0.01 and relative to control group. *p < 0.05 and **p < 0.01 relative to the MK-801-induced model

From Fig. 2a, it can be seen that the mean latency of rats to find the platform declined progressively during the 5 days of training in the Morris water maze test. Compared with the control group, the MK-801-induced rats spent more time to find the platform from the first day to fifth day (*p* < 0.05). The results revealed that MK-801-induced rats showed remarkably cognitive impairment. However, the MK-801 cognitive impairment was improved by treatment with clozapine and PQQ. Figure 2b illustrates the swim paths of rats on the second day and the fourth day. Rats tended to explore all four quadrants of the pool on the second day. On the fourth day, the control rats almost swam directly to the platform, whereas the MK-801-induced rats took longer swimming track. Compared with the MK-801 group, the arts in the clozapine group and the clozapine+PQQ group had less swimming tracks. In the probe test, as shown in Fig. 2c, rats in the MK-801 group exhibited significant shorter time spent in the target quadrant compared with control group (*P*<0.05). Fortunately, this shortened time by MK-801 was greatly prolonged after treatment with PQQ and clozapine (*p* < 0.05). We also measured the crossing times (Fig. 2d), the MK-801-induced rats had less crossing times compared to the control rats (*p* < 0.05). Nevertheless, the rats treated with clozapine and PQQ had more crossing times (*p* < 0.05). These results suggested that clozapine combine with PQQ could improve the learning and memory ability in MK-801-induced schizophrenia rat models.

### Open-field test

**Fig. 3:**
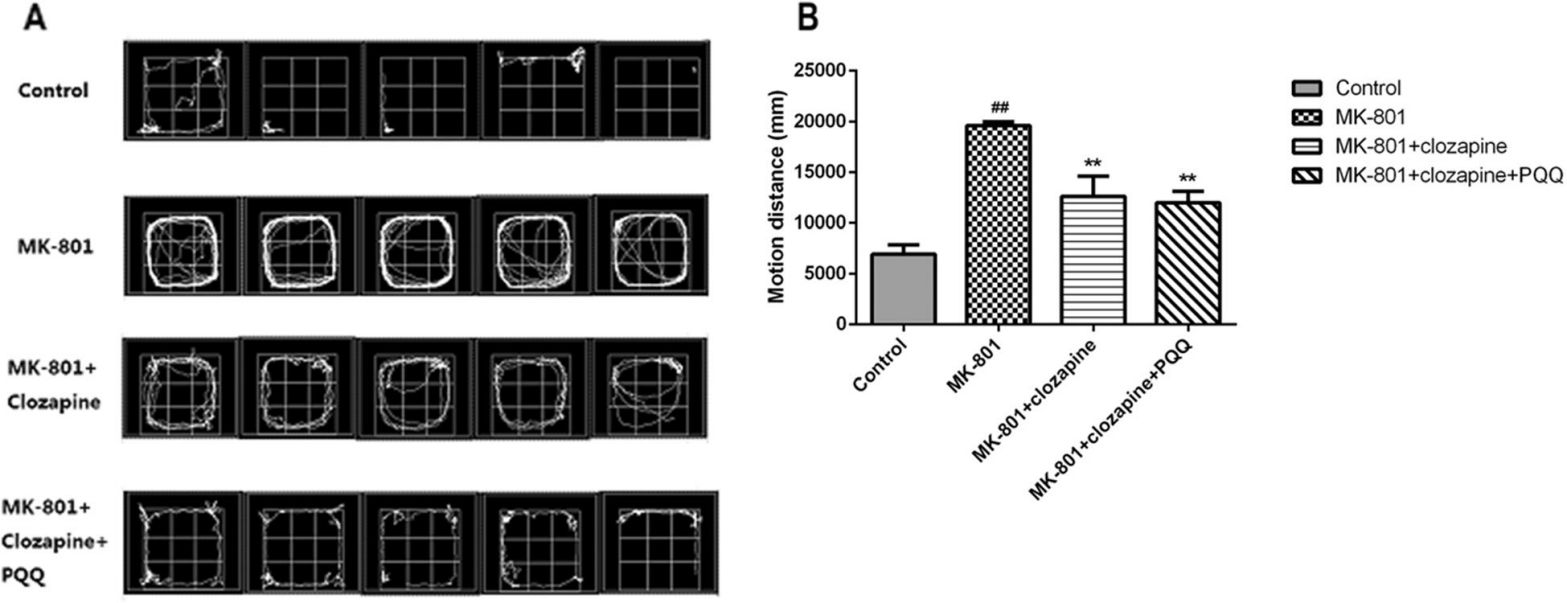
Locomotor activity in the open field test. **a** The trajectory was evaluated 5 tests per 3 min in 15 min. **b** The motion distance was evaluated in the open field. ^#^*p* < 0.05 and ^##^*p* < 0.01 relative to control group. *p < 0.05 and **p < 0.01 relative to the MK-801-induced model

The open-field test was used to evaluate exercise ability and behavioral changes before and after administration. From the trajectory of motion (Fig. 3a), the rats in the MK-801 group had more continuous turn-around behavior and locus of movement compared with the control group. The trajectories of clozapine group and clozapine +PQQ group were reduced compared with the MK-801 group. From the moving distance (Fig. 3b), the rats in the MK-801 group was significantly longer compared with the control group (*P* < 0.01). However, the clozapine group and the clozapine+PQQ group can reduce the distance of movement in rats compared with the MK-801 group (*P* < 0.01). These results suggested that clozapine+PQQ could alleviate anxiety and fear caused by MK-801 and enhance the adaptability of experimental animals to new environments.

### Effects of PQQ and clozapine on Akt phosphorylation

**Fig. 4:**
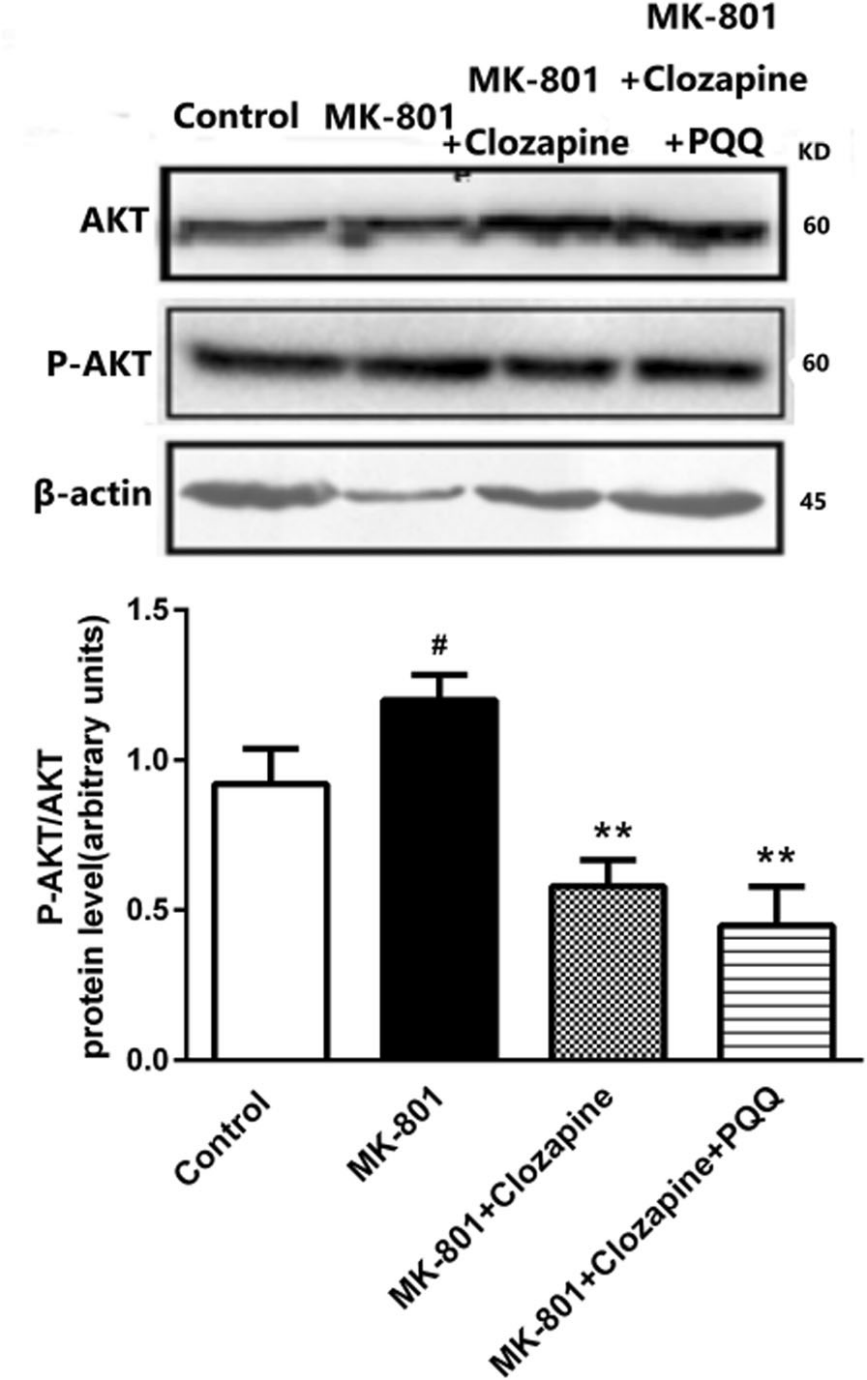
Western blot analysis the protein expression Akt、P-Akt in the hippocampus of rats. β-actin as internal reference of total protein. vs the control group. ^#^*p* < 0.05 and ^##^*p* < 0.01 relative to control group. *p < 0.05 and **p < 0.01 relative to the MK-801-induced model

It has been previously reported that the Akt signaling pathway participates in the therapeutic effects of antipsychotic drugs [28]. Accordingly, we assessed the effects of PQQ and clozapine on Akt phosphorylation. As shown in Fig. 4, treatment with MK-801 increased Akt phosphorylation compared to control rats. Interestingly, clozapine significantly reduced Akt phosphorylation in the hippocampus of rats as compared to MK801 treatment rats. Furthermore, the phosphorylation of Akt in rats treated with clozapine and PQQ decreased more significantly.

### Akt/GSK3β signaling pathway

**Fig. 5:**
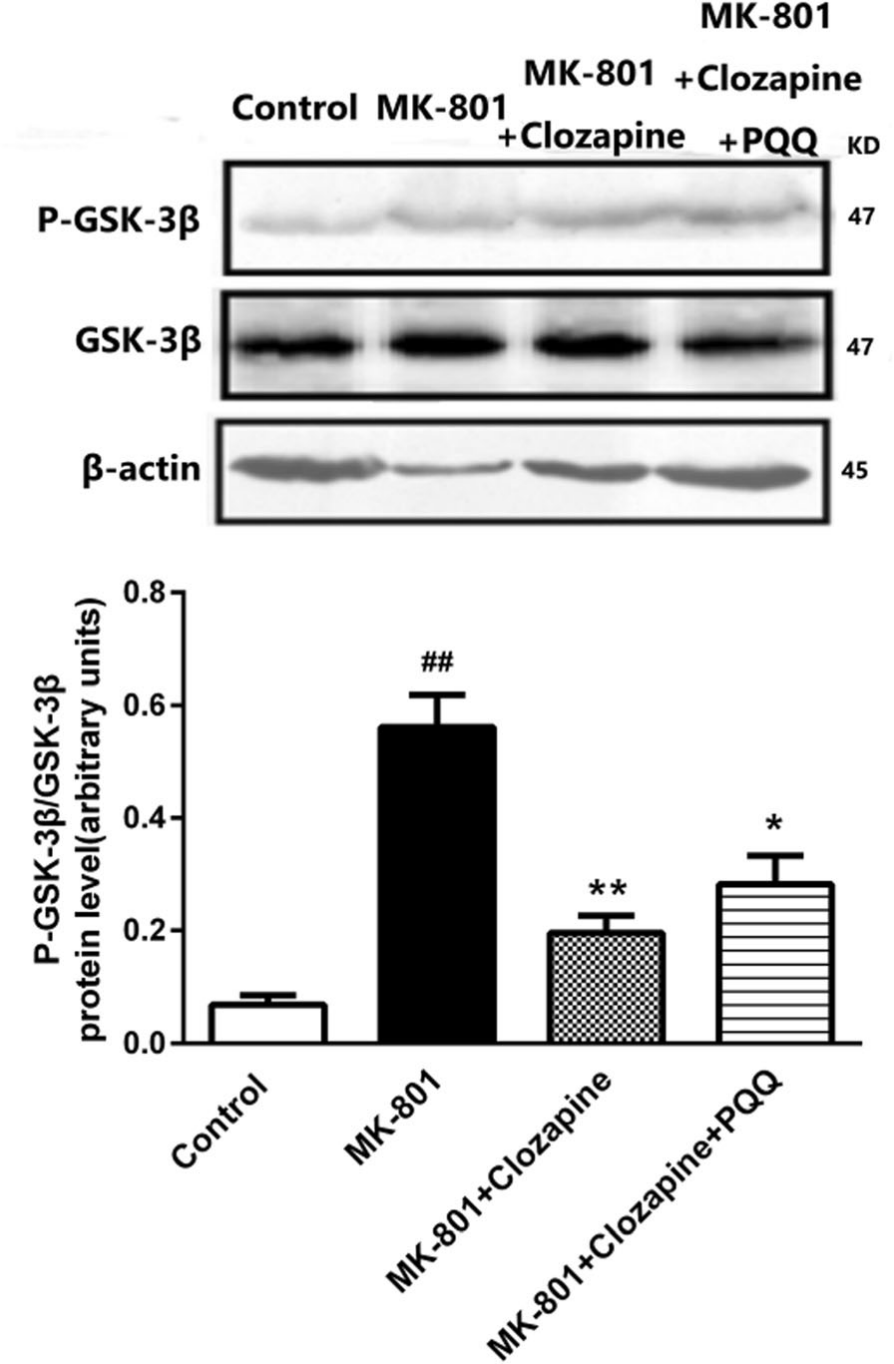
Western blot analysis the protein expression GSK-3β、P-GSK-3β in the hippocampus of rats. β-actin as internal reference of total protein. ^#^*p* < 0.05 and ^##^*p* < 0.01 relative to control group. *p < 0.05 and **p < 0.01 relative to the MK-801-induced model

Antipsychotic drugs may affect Akt/GSK-3β pathway through some receptors acting as antagonists, thus affecting cognitive function. We, therefore, set out to investigate whether the Akt/GSK-3β signaling pathway was involved in atypical antipsychotic drug clozapine combined with PQQ. As indicated in Fig. 5, the level phosphorylation of GSK-3β is dramatically increased in MK-801 rats, and clozapine is able to reduce the increased p-GSK-3β activity. Western blot analysis also verified the inhibition of GSK-3β activity because the phosphorylation level is restored by clozapine combined with PQQ treatment. Meanwhile, clozapine combined with PQQ treatment significantly inhibited the activity of p-Akt. Based on these results, it seems that PQQ combined with clozapine exerts its beneficial effects on MK801-induced cognition deficit through Akt/GSK-3β signaling pathway.

### PQQ combine clozapine prevented apoptosis in MK-801 treated rats

**Fig. 6:**
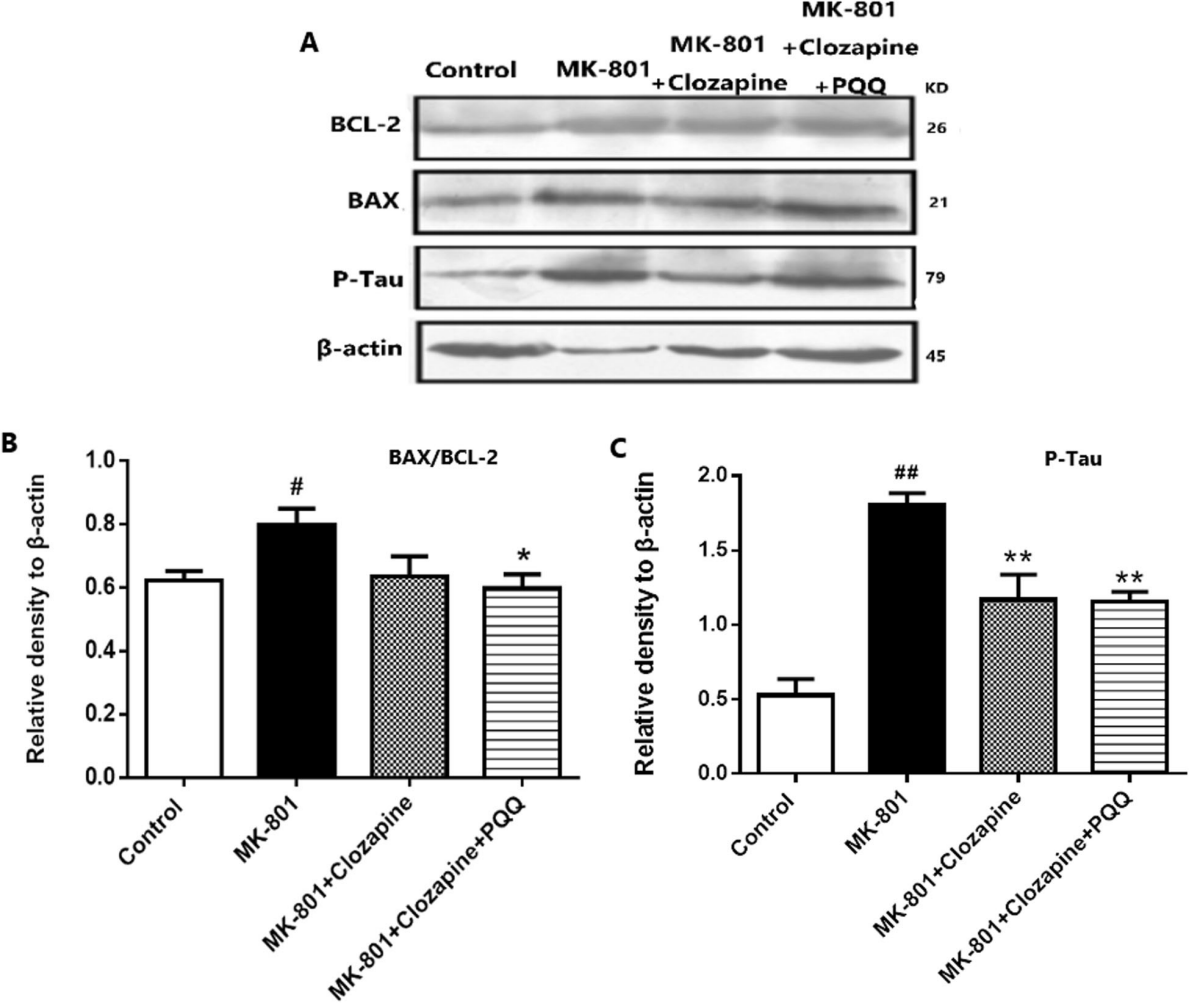
Western blot (**a**) analysis the protein expression of BAX/BCL-2 (**b**) and Tau phosphorylation (**c**) in the hippocampus of rats. β-actin as internal reference of total protein. ^#^*p* < 0.05 and ^##^*p* < 0.01 relative to control group. *p < 0.05 and **p < 0.01 relative to the MK-801-induced model

Bcl-2 and Bax are cytoplasmic proteins that are involved in the regulation of apoptosis. Bcl-2 proteins prevent apoptotic death in neurons, while Bax proteins cause apoptosis by damaging mitochondria. To investigate the effect of PQQ and clozapine on the expression level of Bax and Bcl2, we performed western blot analysis and determined the ratio of Bax/Bcl2. Our results confirmed that MK-801 treatment significantly increased the ratio of Bax/Bcl2 proteins in the MK-801-treated rats as compared to the control group. Interestingly, PQQ combine clozapine reversed and significantly decreased the MK-801-induced Bax/ Bcl2 (*P* < 0.001), showing anti-apoptotic effect (Fig. 6).

Tau is an important microtubule-associated protein that promotes the assembly and stabilization of microtubules. Hyperphosphorylated tau reduces its ability of stabilizing microtubule and leads to disruption of the cytoskeletal arrangement, which is another major pathology involved in cognitive deficits in neurological diseases. Moreover, some studies reported that tau is hyperphosphorylated in the hippocampus of long-term-estrogen-deprivation rats. In this work, by western blotting, we found that phosphorylated tau increased by 2.4 times in hippocampus of MK-801 rats compared with the control rats (Fig. 6, *P* < 0.01). Fortunately, clozapine combined with PQQ significantly reduced phosphorylated tau, as clozapine did.

All these data suggested that clozapine+PQQ improved the spatial cognition deficit of MK-801-induced rats, partly by decreasing hippocampal tau hyperphosphorylation and inhibiting apoptosis.

### The effect of PQQ and clozapine on the expression of glutamate receptor

**Fig. 7:**
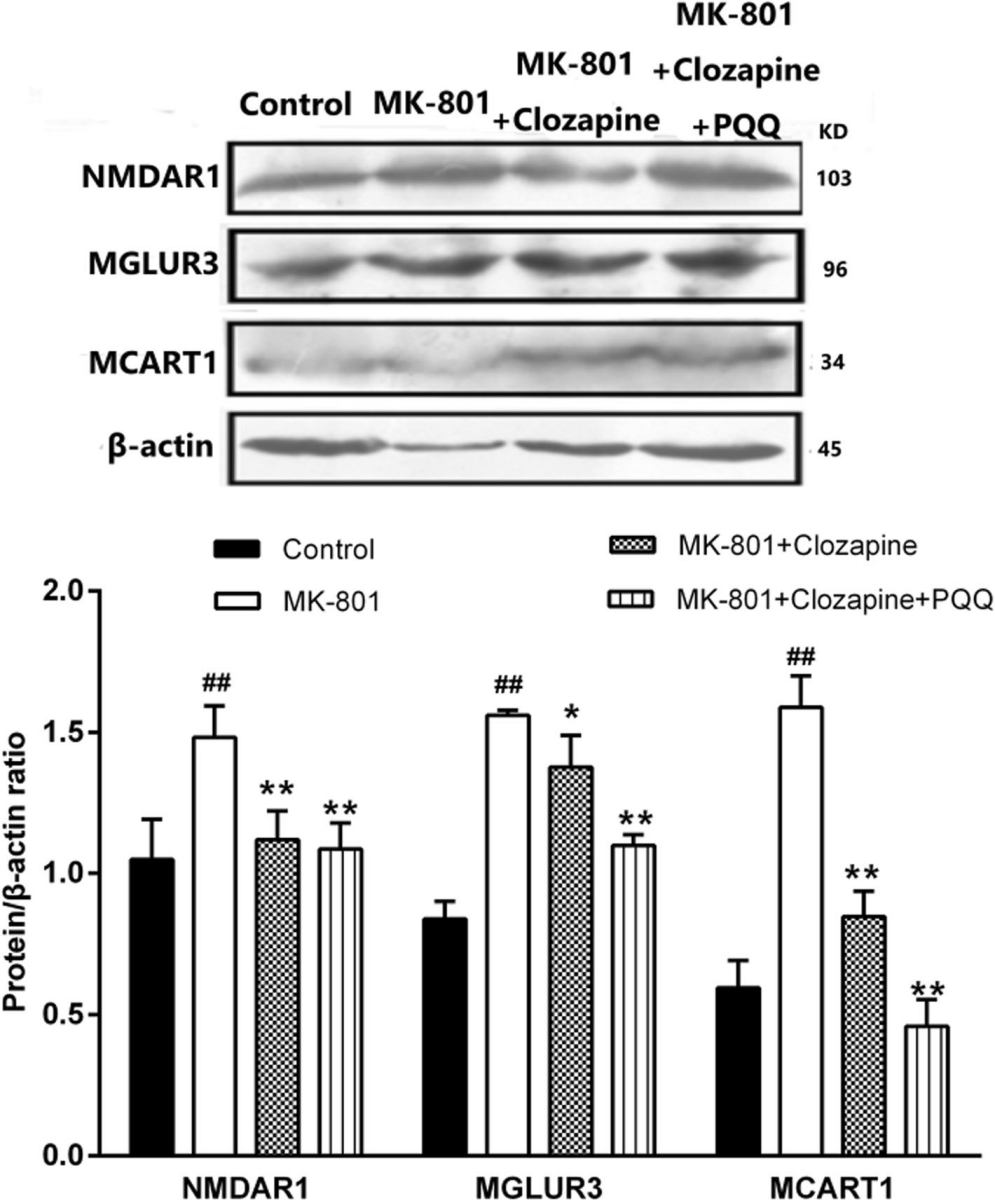
Western blot analysis the protein expression of NMDAR1 and MGLUR3 in the hippocampus of rats. β-actin as internal reference of total protein. ^#^*p* < 0.05 and ^##^*p* < 0.01 relative to control group. *p < 0.05 and **p < 0.01 relative to the MK-801-induced model

Glutamate dysfunction has strongly been implicated in the etiology of schizophrenia, particularly a hypofunction of the NMDAR. In this work, we studied the expression of MGLUR1, MGLUR2, MGLUR3 and NMDAR1 receptors in hippocampus and the effects of PQQ and clozapine on them. As shown in Fig. 7, we found that the expressions of MGLUR1 and NMDAR1 in the hippocampus of MK801 rats increased significantly compared with the control group. Clozapine can reduce the protein expression levels of NMDAR1、MGLUR3 respectively (*P*<0.01 and *P*<0.05). The protein expression levels of NMDAR1 and MGLUR3 were significantly decreased after combined administration of clozapine and PQQ (*P* < 0.01). However, MGLUR2 and MGLUR3 were not detected.

## Discussion

Cognitive impairment is a major feature of schizophrenia, which has been extensively studied. However, there is still no effective treatment for people diagnosed with schizophrenia [29]. The etiology of cognitive impairment in schizophrenia is complex and remains unclear. The aim of this study was to investigate the protective effect of PQQ combined with clozapine on cognitive function in MK801-induced schizophrenic rats and the possible underlying mechanisms. We found that clozapine combining with PQQ treatments could ameliorate the memory deficits in MK-801 induced schizophrenia rats partially by reducing the expression of NMDAR1 and MGLUR3, decreasing hippocampal tau hyperphosphorylation and inhibiting apoptosis through GSK-3β/Akt signaling pathway. Co-agonist NMDA receptor can produce additive NMDA function regulation, which provides a new experimental basis for the treatment of people diagnosed with schizophrenia.

GSK-3β plays a significant role in cognitive function of schizophrenia. It has been shown that reduced GSK-3β expression in postmortem prefrontal cortex of schizophrenic patients may be an etiological factor of this disorder [30]. Accumulating evidence suggests that GSK-3β inhibits hippocampal neurogenesis and other processes related to learning and memory [31].

Aligned with this, evidence demonstrate that inhibiting the GSK-3β over-expression can significantly reduce memory deficits [32, 33]. At the same time, convergent evidence shows that the expression of GSK-3β and its related signaling pathways have changed in individuals affected by schizophrenia [34]. In addition, several different atypical antipsychotic drugs have been reported to increase the inhibitory phosphorylation of GSK-3β in brain regions of rats [35–38]. Furthermore, some other studies have reported a high correlation between Akt gene variants and schizophrenia [39, 40]. Emamian also showed convincing evidence that Akt/GSK-3β signal was severely impaired in schizophrenic patients [41].

Several different atypical antipsychotic drugs have been reported to increase the inhibitory phosphorylation of GSK-3β in brain regions of rats [39] . In this experiment, we found MK801 up-regulated the expression of GSK-3β and AKT in varying degrees in the hippocampus, especially p-GSK3β. Fortunately, the expression of p-GSK3β and p-Akt was markedly reduced by PQQ combined with clozapine, resulting in down-regulation of p-Tau level in hippocampus. However, the corresponding changes were not found in other parts of the brain, such as the cortex and cerebellum. Hippocampus is integral to memory formation, and Hippocampal dysfunction is involved in cognitive disruption in individuals affected by schizophrenia [42].

Although previous studies have confirmed that GSK-3β inhibition may be an effective strategy to improve cognitive impairment in schizophrenic patients, it should be noted that all antipsychotic drugs currently used clinically are antagonists against D2 receptors. However, antipsychotic drugs targeting D2 receptors were ineffective in cognitive therapy for schizophrenia [43]. The pathophysiology of schizophrenia is complex. It was the demonstration that NMDAR antagonists could replicate the full range of psychotic, negative, cognitive, and physiologic features of schizophrenia. Other evidence suggests that some drugs that enhance NMDAR function can significantly improve cognitive function in schizophrenia [44]. Thus, combining GSK-3 inhibition strategy with other targets such as NMDAR antagonists crucial to cognition might be a more useful approach for the development of more effective treatments for people diagnosed with schizophrenia.

In this study, MK801 was used to establish a model of NMDA receptor hypofunction in schizophrenia. Our aim is to regulate the impaired GSK signaling pathway induced by MK801 via co-antagonizing NMDA receptor in the treatment of cognitive impairment in schizophrenia. Our results showed that MK801-treated rats exhibited positive symptoms of schizophrenia with locomotor hyperactivity and stereotyped behavior. Rats treated with clozapine and PQQ were restored in locomotor hyperactivity, stereotyped behavior and cognitive function. Western blot results showed that combination of PQQ with clozapine treatment rescues cognition deficit via the Akt /GSK-3β signal pathway.

It has been reported that MGLURs could be valuable targets for antipsychotic compounds. NMDAR1 and MGLUR3 have been found to act significantly reverse glutamate efflux and to attenuate behavioral and cognitive effects. Our results showed that the protein expression levels of NMDAR1 and MGLUR3 in hippocampus were significantly decreased after clozapine combined with PQQ. However, other limitations that may shape interpretation of these findings should be considered. Reverse cognitive impairment have different sensitivity to NMDA antagonism in different species [45]. To support these findings, more species need to be studied in the next experiment.

## Conclusions

In summary, our results showed that clozapine combining with PQQ treatments could ameliorate the memory deficits in MK-801 induced schizophrenia rats partially by reducing the expression of NMDAR1 and MGLUR3, decreasing hippocampal tau hyperphosphorylation and inhibiting apoptosis through Akt/GSK-3β signaling pathway. These data suggest that clozapine combining with PQQ treatments may be clinically potential and usable agents for individuals affected by schizophrenia. More work remains to be done before we understand the exact mechanism.

## Data Availability

The datasets analyzed during the current study are not publicly available but are available from the corresponding author on reasonable request.

## Abbreviations

Akt: Protein kinase B
GSK-3β: Glycogen Synthase Kinase-3β
MGLUR: Metabotropicglutamate receptor
MWM: Morris water maze test
NMDAR: N-methyl-D-aspartate receptor
PQQ: Pyrroloquinoline quinone
